# With the leisure of time, kinetic proofreading can still perform reliable ligand discrimination

**DOI:** 10.1073/pnas.2320118121

**Published:** 2024-07-25

**Authors:** Fangzhou Xiao, Vahe Galstyan

**Affiliations:** ^a^Department of Physics, University of California, San Diego, La Jolla, 92093 CA; ^b^Center for Living Systems, AMOLF, Science Park 104, Amsterdam 1098 XG, The Netherlands

Kinetic proofreading is a canonical scheme believed to be responsible for the high ligand discrimination capacity of many biochemical processes. In a recent PNAS publication, Kirby and Zilman studied kinetic proofreading for receptor signaling and argued that when stochasticity is taken into account, having more proofreading steps does not generally improve the discrimination of different receptor-binding ligands based on counts of signaling molecules produced (1).

**Fig. 1.**
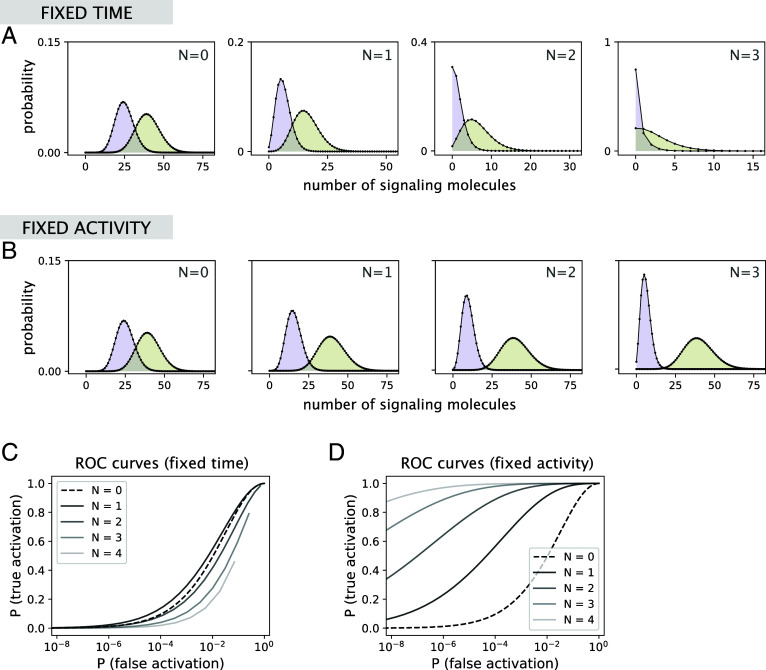
Discrimination performance can exhibit diverse responses to varying the number of proofreading steps. (*A*) Signal distributions arising from the binding of cognate (lower *k*_off_) and noncognate (higher *k*_off_) ligands for different choices of the number of proofreading steps *N* in the fixed time setting. Parameter used are the same as in figure 2B of Kirby and Zilman: $k_{\mathrm{on}}c/k_{p}=1$, $k_{f}/k_{p}=1$, $k_{p}t=100$, $k_{\mathrm{off},1}/k_{p}=3$, and $k_{\mathrm{off},2}/k_{p}=1.5$. Distributions are obtained from solving the full master equation in Eq. **11** of Kirby and Zilman (see ref. 5 for the code). (*B*) Signal distributions for different choices of *N* in the fixed activity setting where discrimination time *t*_*N*_ scales with *N* as $t_{N}=t_{0}{\left(1+k_{\mathrm{off},2}/k_{f}\right)}^{N}$ with $k_{p}t_{0}=100$. All other parameters are the same as in panel (*A*). (*C*) ROC curves for different choices of *N* corresponding to panel (*A*). The true activation probability for a given threshold $n^{*}$ is computed as $P(n>n^{*}|k_{\mathrm{off},2})$, and the false activation probability is computed as $P(n>n^{*}|k_{\mathrm{off},1})$. (*D*) ROC curves corresponding to the fixed activity setting in panel (*B*).

In their analysis, however, the authors made an implicit assumption about a fixed discrimination time, i.e., the time to produce signaling molecules before the cell is asked to discriminate is kept the same when the number of proofreading steps *N* is varied. Indeed, in such a setup, the distributions of signaling molecule counts arising from cognate (lower *k*_off_) and noncognate (higher *k*_off_) ligand binding tend to overlap more with increasing *N* (Fig. 1*A*), which results in decreasing discrimination performance, as measured by the probability of false activation (Fig. 1*C*). This assumption, however, is not always warranted, since discrimination time could naturally change with *N* due to feedback (2) or signaling threshold mechanisms (3, 4).

Here, we show that when the discrimination time is allowed to vary, the proofreading performance can, in fact, uniformly improve with the number of steps. Specifically, we consider a fixed activity setup where discrimination time increases with *N* to maintain the same mean level of signaling molecules produced from cognate ligand binding. This is a biologically motivated scenario where a fixed threshold of signaling activity is required for downstream decision-making. While the signaling molecule count distribution corresponding to cognate ligand binding remains mostly unchanged as *N* is increased, the distribution corresponding to noncognate ligand binding uniformly drifts toward zero, reducing the overlap between the two distributions (Fig. 1*B*). As a result, discrimination performance improves with *N* (Fig. 1*D*), which is the opposite of the fixed time behavior considered by Kirby and Zilman.

While fixing signaling activity is a natural choice for thresholding-based schemes, it is arguable that fixing the discrimination time could also be biologically relevant when there is a time constraint for eliciting cellular response. Kirby and Zilman’s work therefore opens an avenue for a comprehensive study of the interplay between discrimination time, discrimination accuracy, and signaling activity, where the two cases discussed here will be slices of the trade-off surface along different axes.
